# Comparison between reference values for FVC, FEV_1_, and FEV_1_/FVC ratio in White adults in Brazil and those suggested by the Global Lung Function Initiative 2012*

**DOI:** 10.1590/S1806-37132014000400007

**Published:** 2014

**Authors:** Carlos Alberto de Castro Pereira, Andrezza Araujo Oliveira Duarte, Andrea Gimenez, Maria Raquel Soares

**Affiliations:** Graduate Program, Federal University of São Paulo; and Physician. Pulmonary Function Laboratory, Centro Diagnóstico Brasil, São Paulo, Brazil; Federal University of Campina Grande, Campina Grande, Brazil; Pulmonary Function Laboratory, Centro Diagnóstico Brasil, São Paulo, Brazil; Pulmonary Function Laboratory, Centro Diagnóstico Brasil, São Paulo, Brazil

**Keywords:** Respiratory function tests/statistics and numerical data, Respiratory function tests/diagnosis, Reference values

## Abstract

**OBJECTIVE:**

To evaluate the spirometry values predicted by the 2012 Global Lung Function Initiative (GLI) equations, which are recommended for international use, in comparison with those obtained for a sample of White adults used for the establishment of reference equations for spirometry in Brazil.

**METHODS:**

The sample comprised 270 and 373 healthy males and females, respectively. The mean differences between the values found in this sample and the predicted values calculated from the GLI equations for FVC, FEV_1_, and VEF_1_/FVC, as well as their lower limits, were compared by paired t-test. The predicted values by each pair of equations were compared in various combinations of age and height.

**RESULTS:**

For the males in our study sample, the values obtained for all of the variables studied were significantly higher than those predicted by the GLI equations (p < 0.01 for all). These differences become more evident in subjects who were shorter in stature and older. For the females in our study sample, only the lower limit of the FEV_1_/FVC ratio was significantly higher than that predicted by the GLI equation.

**CONCLUSIONS:**

The predicted values suggested by the GLI equations for White adults were significantly lower than those used as reference values for males in Brazil. For both genders, the lower limit of the FEV_1_/FVC ratio is significantly lower than that predicted by the GLI equations.

## Introduction

The interpretation of pulmonary function tests is based on comparisons between data obtained for an individual patient and (predicted) reference values derived from healthy subjects. Ideally, reference values should be derived from a population similar to that tested, using appropriate equipment and following standard procedures.^(^ 1 ^)^

Pulmonary function values differ substantially among different regions of the world, which has been attributed to anthropometric, environmental, social, and genetic factors, as well as to technical factors.^(^ 1 ^-^ 4 ^)^ Attempts to compile equations by different authors were made for Europe in 1983^(^ 5 ^)^ and again in 1993.^(^ 6 ^)^ Those recommendations of the work group were accepted and made official by the European Respiratory Society (ERS), which supported their widespread use in Europe.

In 2005, a joint guideline of the American Thoracic Society (ATS) and the ERS recommended that the equations derived in the Third National Health and Nutrition Examination Survey (NHANES III) be adopted in the USA, but it did not endorse the use of equations for Europe, recommending that new reference values be obtained.^(^ 1 ^)^ The latter recommendation was based on the fact that the derivation of reference values for spirometry in various European countries after 1993 demonstrated that the equations proposed by Quanjer et al. underestimated predicted values. ^(^ 6 ^-^ 10 ^)^ This finding was confirmed by various studies published after 2005.^(^ 11 ^-^ 15 ^)^ Similar results were observed when reference values derived for the Brazilian population were compared with those proposed by Quanjer et al.^(^ 6 ^,^ 16 ^,^ 17 ^)^

Various limitations were identified in the derivation of the equations that were compiled by that group of authors, and it was suggested that those reference values be abandoned,^(^ 18 ^)^ although studies using those reference values continue to be published.

In 2012, an even bolder proposal was suggested by Quanjer et al.: the derivation of universal equations.^(^ 19 ^)^ Data on reference values derived from 72 centers in 33 countries were provided for the derivation of the equations. In Latin America, values derived in the *Projeto Latino-Americano de Investigação em Obstrução Pulmonar* (PLATINO, Latin American Project for the Investigation of Obstructive Lung Disease), which included subjects over 40 years of age, were provided.^(^ 20 ^)^ We decided not to send the equations derived for adults in the Brazilian population because of the limitations observed in the previous study by Quanjer et al.^6)^ and because we do not believe that a universal pulmonary function equation is possible. The proponents of the universal equation acknowledge that the included data from Latin America are scarce and that that equation should not be used in the continent.

However, values for White adults were suggested, and we tested the hypothesis that those values could fit our population.

## Methods

The predicted values derived from the ERS Global Lung Function Initiative (GLI) equations^(^ 19 ^,^ 21 ^)^ for White adults were calculated for males and females by using data on gender, height, and age found in a study of reference values for the Brazilian population.^(^ 16 ^)^ The patients selected completed a standard respiratory questionnaire,^(^ 22 ^)^ were nonsmokers, had no respiratory symptoms, and had no cardiopulmonary disease. The Brazilian sample included 270 males (age, 25-86 years; height, 152-192 cm) and 373 females (age, 20-85 years; height, 137-182 cm).

The equations derived for males were as follows^(^ 16 ^)^:

*FVC* = *H* × 0.0517 − *A* × 0.0207 - 3.18 (lower limit of normality [LLN] = −0.90) 

*FEV1* = *H* × 0.0338 − *A* × 0.0252 - 0.789 (LLN = −0.76) 

*FEV1/FVC* × 100 = 120.3 − *H* × 0.175 − *A* × 0.197 (LLN = −7.6) 

where *H* is height in cm and *A* is age in years.

The equations derived for females were as follows^(^ 16 ^)^: 

*FVC* = *H* × 0.041 − *A* × 0.0189 - 2.848 (LLN = −0.64) 

*FEV1* = *H* × 0.0314 − *A* × 0.0203 - 1.353 (LLN = −0.61) 

*FEV1/FVC* × 100 = 111.5− *H* × 0.140 − *A* × 0.158 (LLN = −8.3) 

The GLI equation used to derive the parameter values is as follows:

log(*Y*) = 5*a* + *b* × log(*H*) + *c* × log(*A*) + *AS* + *d* × group 

where *Y* is the dependent variable, *H* is height in cm, *A* is age in years, and *AS* is age spline.

Group takes a value of 1 for White adults, and this value was used in the present study. The Brazilian equation for FVC and FEV_1_ is linear:

*Y* = *a* × *H* − *b* × *A* - constant 

The mean values found in the Brazilian sample for FVC, FEV_1_, and FEV_1_/FVC, as well as their lower limits, were compared with the predicted values calculated from the GLI equations on the basis of the age and height of individual subjects in the Brazilian sample. Paired t-test was used for the comparisons.

Subsequently, on the basis of the Brazilian values, a linear regression analysis was performed between age (independent variable) and height (dependent variable). Regression equations were used to calculate the expected value for height at ages 25, 50, and 75 years for both genders. The values calculated from the GLI equation and those calculated from the Brazilian equation were tabulated and compared in various combinations of age and height.

All statistical procedures were performed with the IBM SPSS Statistics software, version 20.0 (IBM Corp., Armonk, NY, USA).

## Results

The mean differences between the predicted values calculated from the Brazilian equations and those generated by the GLI equations, as well as their lower limits, are shown in Table 1. For males, the values found in the Brazilian sample for all of the variables studied were significantly higher than those generated by the GLI equations. For females, there were practically no differences, except for the lower limit of the FEV_1_/FVC ratio, for which the values in the Brazilian sample were significantly higher than those generated by the GLI equations.

**Table 1:** Mean differences for the variables studied, calculated by subtracting the predicted values found in the Brazilian population(16) from those generated by the Global Lung Function Initiative equations(19,21), by gender.a

Variable	Gender					
	Male			Female		
	Δ	t	p	Δ	t	p
FVC	0.29 ± 0.62	7.81	< 0.001	−0.01 ± 0.38	−0.75	0.46
LL	0.30 ± 0.59	9.41	< 0.001	0.01 ± 0.38	0.65	0.52
FEV_1_	0.28 ± 0.50	9.06	< 0.001	0.00 ± 0.33	0.36	0.72
LL	0.29 ± 0.48	10.12	< 0.001	−0.02 ± 0.33	−0.93	0.36
FEV_1_/FVC	0.93 ± 4.89	3.14	0.002	0.02 ± 5.00	0.06	0.95
LL	3.27 ± 4.71	11.43	< 0.001	3.68 ± 5.23	13.55	< 0.001

LL : lower limit

a Values expressed as mean ± SD.

When the data tabulated for the various combinations of age and height were compared, additional data could be observed (Tables 2 and 3). For males, the differences were more evident in shorter, older subjects. In subjects aged 75 years, the differences for FVC and for its lower limit were 0.36 L and 0.38 L, respectively. For these same subjects, the difference for FEV_1_ and for its lower limit was 0.29 L for both.

It is also of note that the FEV_1_/FVC ratio was lower as calculated from the GLI equation, with the difference increasing with age.

**Table 2:** Predicted spirometry values for the Brazilian population(16) and those generated by the Global Lung Function Initiative (GLI) equation(19,21) for combinations of age and height in males.

Variable	Age, years	Height, cm	Pereira et al.^(16)^	GLI^(19,21)^
FVC, L	25	175	5.35	5.18
	50	170	4.58	4.48
	75	165	3.80	3.44
LL	25	175	4.45	4.19
	50	170	3.68	3.50
	75	165	2.90	2.52
FEV_1_, L	25	175	4.50	4.35
	50	170	3.69	3.56
	75	165	2.90	2.61
LL	25	175	3.74	3.51
	50	170	2.93	2.78
	75	165	2.14	1.85
FEV_1_/FVC	25	175	0.85	0.85
	50	170	0.81	0.80
	75	165	0.77	0.76
LL	25	175	0.77	0.73
	50	170	0.73	0.69
	75	165	0.69	0.62

LL : lower limit.

**Table 3:** Comparison between predicted spirometry values for the Brazilian population(16) and those generated by the Global Lung Function Initiative (GLI) equation(19,21) for combinations of age and height in females.

Variable	Age, years	Height, cm	Pereira et al.^(16)^	GLI^(19,21)^
FVC, L	25	162	3.82	3.84
	50	158	3.18	3.24
	75	153	2.47	2.31
LL	25	162	3.18	3.07
	50	158	2.54	2.53
	75	153	1.83	1.63
FEV_1_, L	25	162	3.23	3.30
	50	158	2.60	2.60
	75	153	1.90	1.79
LL	25	162	2.62	2.65
	50	158	1.93	2.03
	75	153	1.32	1.28
FEV_1_/FVC	25	162	0.85	0.87
	50	158	0.81	0.81
	75	153	0.78	0.78
LL	25	162	0.77	0.75
	50	158	0.73	0.70
	75	158	0.68	0.64

LL : lower limit.

## Discussion

In the present study, universal reference equations for spirometry proved unable to predict spirometry values in the Brazilian population accurately.

Various reference value equations have been published in recent decades. The expected values for individuals with a certain combination of age and height can differ considerably.^(^ 1 ^-^ 3 ^)^

Such variations can be explained by the criteria used for selecting 'normal' populations, by the equipment used, by the measurement techniques, by the biological variability of populations, by socioeconomic and environmental factors, and by the statistical models used in the data analysis.

In 2005, the ATS and ERS published a joint guideline on pulmonary function.^(^ 1 ^)^ Reference values were suggested for children and adults in the United States; however, values for other places remained to be established. As a result of this lack of recommendation, a group of authors, led by Quanjer, founded the GLI in Berlin in 2008. In April of 2010, the group received, as occurred previously,^(^ 5 ^,^ 6 ^)^ the seal of the ERS as a task force.^(^ 19 ^)^ In 2012, values derived from data sent from various places were grouped, as occurred with European data in 1993,^(^ 6 ^)^ and reference values for subjects aged 3-95 years were suggested. In total, 74,187 nonsmokers from 26 countries in five continents were included in equations derived by combining various studies. The data relating to South America, which were derived from a study conducted in Latin America^(^ 20 ^)^ and from a sample of children in Mexico,^(^ 23 ^)^ were disregarded because of differences in height and in predicted values, as well as because of the lack of data for subjects aged 25-40 years. However, according to the published supplement, 178 cases of White adults in Brazil were included.^(^ 19 ^)^

The values for White adults were derived especially from five large studies: two conducted in the United States^(^ 24 ^,^ 25 ^)^ and three conducted in Europe.^(^ 7 ^,^ 10 ^,^ 13 ^)^ It is of note that the values derived in those studies differ, which was attributed to the different equipment used. However, various factors, such as sample selection, measurement techniques, and quality control, also influence the results obtained, which complicates the aggregation of different studies.

Comparing the values calculated from the GLI equation with the data derived from a sample used for the establishment of reference equations for spirometry in Brazil,^(^ 16 ^)^ we found that, for males, the use of the GLI equation results in lower values both in terms of predicted values and of their lower limits. For females, the values are quite similar, except for the FEV_1_/FVC ratio and its lower limit, for which the values in the Brazilian sample are higher than those generated by the GLI equation. These findings indicate that the use of the GLI equation will fail to diagnose reductions in FVC and, therefore, will have lower sensitivity in detecting obstructive lung disease in males. For both genders, the sensitivity for the diagnosis of obstructive lung disease will be lower with the use of the GLI equations, given that the lower limit of the FEV_1_/FVC ratio as calculated from these equations is significantly lower, especially in older subjects.

The differences between the predicted values calculated from the GLI equation for the FEV_1_/FVC ratio and its lower limits vary because of the regression model used; however, they are, on average, 0.11 for males and 0.12 for females,^(^ 21 ^)^ which exceeds the values derived in Brazil (0.08 for males and 0.09 for females).^(^ 16 ^)^

Recent studies have compared spirometric diagnosis by the equation suggested by the GLI and by other equations. One study compared spirometric diagnosis by three equations in 17,572 tests (subjects aged 18-85 years) performed in laboratories in Australia and Poland.^(^ 26 ^)^ The values calculated from the equations derived by the GLI were higher than those calculated from the equations derived by Quanjer et al.,^(^ 6 ^)^ as expected. Differences in the lower limits resulted in a significant reduction in the diagnosis of restrictive lung disease when the GLI equation was compared with the NHANES III equation, although the latter was incorporated into the GLI equation (but comprised less than 4% of the sample). In males, restrictive lung disease was diagnosed in 22.6% by the NHANES III equation and in 17.1% by the GLI equation. In females, the proportions were 22.8% and 8.1%, respectively.

In a study conducted in Tunisia, local predicted values and those suggested by the GLI were used in 1,192 consecutive spirometries in adults aged 18-60 years.^(^ 27 ^)^ Again, the proportion of cases diagnosed with restrictive lung disease by the use of the local equation (19.0%) was greater than that diagnosed by the GLI equation (8.4%).

The findings of the aforementioned studies are not surprising, given the wide range for determination of lower limits by the GLI equation, which is the result of the combination of several equations for which quality control and results were different.

In conclusion, the values suggested by the multiethnic reference equation proposed by the GLI, developed for White adults, differ significantly from the values derived for White adult males in Brazil. For females, the values derived are similar for FVC, FEV_1_, and their lower limits. For both genders, the lower limit of the FEV_1_/FVC ratio is significantly lower as calculated from the GLI equation.
