# Supplementary material for: Comparison between reference values for FVC, FEV1, and FEV1/FVC ratio in White adults in Brazil and those suggested by the Global Lung Function Initiative 2012
Source: J Bras Pneumol. 2014 Jul-Aug;40(4):397–402. doi: 10.1590/S1806-37132014000400007 (PMC4201170; doi:10.1590/S1806-37132014000400007)
Supplement: Translation

# Translation: Comparação entre os valores de referência para CVF, VEF_1_ e relação VEF_1_/CVF em brasileiros caucasianos adultos e aqueles sugeridos pela Global Lung Function Initiative 2012*

**Authors:** Carlos Alberto de Castro Pereira, Andrezza Araujo Oliveira Duarte, Andrea Gimenez, Maria Raquel Soares

**Affiliations:** Programa de Pós-Graduação, Universidade Federal de São Paulo; e Médico. Laboratório de Função Pulmonar, Centro Diagnóstico Brasil, São Paulo (SP) Brasil; Universidade Federal de Campina Grande, Campina Grande (PB) Brasil; Laboratório de Função Pulmonar, Centro Diagnóstico Brasil, São Paulo (SP) Brasil; Laboratório de Função Pulmonar, Centro Diagnóstico Brasil, São Paulo (SP) Brasil

**Keywords:** Testes de função respiratória/estatística e dados numéricos, Testes de função respiratória/diagnóstico, Valores de referência

## Abstract

**OBJETIVO:**

Comparar os valores espirométricos previstos pelas equações da *Global Lung Function Initiative* (GLI) em 2012, sugeridas como de uso internacional, com aqueles obtidos em uma amostra utilizada para derivação de valores de referência em adultos caucasianos brasileiros.

**MÉTODOS:**

A amostra utilizada era composta por 270 homens e 373 mulheres saudáveis. As médias das diferenças entre os valores dessa amostra e os valores previstos calculados a partir das equações da GLI para CVF, VEF_1_ e VEF_1_/CVF, assim como seus limites inferiores, foram comparados por teste de t pareado. Os valores previstos pelos pares das equações foram comparados em diversas combinações de idade e estatura.

**RESULTADOS:**

Nos homens da amostra, os valores obtidos para todas as variáveis estudadas foram significativamente maiores que aqueles previstos pelas equações da GLI (p < 0,01 para todas). Estas diferenças se tornaram mais evidentes em indivíduos com menor estatura e idade mais avançada. Nas mulheres, somente o limite inferior da relação VEF_1_/CVF foi significativamente maior na amostra brasileira.

**CONCLUSÕES:**

Os valores previstos sugeridos pelas equações da GLI para caucasianos são significativamente menores daqueles utilizados como referência para homens brasileiros. Em ambos os sexos, o limite inferior da relação VEF_1_/CVF é significativamente menor que o previsto pelas equações GLI

## Introdução

A interpretação da função pulmonar é habitualmente baseada em comparações dos dados obtidos de um paciente individual com valores de referência (previstos) derivados de indivíduos saudáveis. Idealmente, os valores de referência devem ser derivados de uma população semelhante à dos indivíduos testados, usando-se equipamentos adequados e procedimentos padronizados.^(^ 1 ^)^

Os valores para a função pulmonar diferem substancialmente entre as diversas regiões do mundo, o que tem sido atribuído a fatores antropométricos, ambientais, sociais e genéticos, assim como a fatores técnicos.^(^ 1 ^-^ 4 ^)^ Tentativas para a compilação de equações de diferentes autores foram realizadas para a Europa em 1983^(^ 5 ^)^ e, novamente, em 1993.^(^ 6 ^)^ Tais recomendações do grupo de trabalho foram aceitas e tornadas oficiais pela *European Respiratory Society* (ERS), com a recomendação para seu uso generalizado na Europa.

Em 2005, uma diretriz conjunta da *American Thoracic Society* (ATS) e da ERS recomendou que as equações derivadas pelo estudo *Third National Health and Nutrition Examination Survey* (NHANES III) fossem adotadas nos EUA, porém não endossou o uso de equações para a Europa, recomendando que novos valores de referência fossem obtidos.^(^ 1 ^)^ Essa recomendação se baseou na derivação de valores de referência para espirometria em diversos países europeus após 1993, os quais demonstraram que as equações propostas por Quanjer et al. subestimavam os valores previstos.^(^ 6 ^-^ 10 ^)^ Esse achado foi confirmado por diversos estudos publicados após 2005.^(^ 11 ^-^ 15 ^)^ Resultados semelhantes foram observados quando valores de referência derivados para a população brasileira foram comparados com os propostos por Quanjer et al.^(^ 6 ^,^ 16 ^,^ 17 ^)^

Diversas limitações foram apontadas na derivação das equações compiladas por aquele grupo de autores e uma sugestão para o abandono daqueles valores de referência foi feita,^(^ 18 ^)^ embora ainda hoje trabalhos sejam publicados com tais valores de referência. 

Em 2012, uma proposta ainda mais ousada foi sugerida por Quanjer et al.: uma derivação de equações universais.^(^ 19 ^)^ Dados de valores de referência derivados de 72 centros de 33 países foram cedidos para derivação das equações. Na América Latina, valores derivados a partir do Projeto Latino-Americano de Investigação em Obstrução Pulmonar (PLATINO), que incluiu indivíduos com mais de 40 anos, foram cedidos.^(^ 20 ^)^ Resolvemos não enviar as equações derivadas para adultos da população brasileira pelas limitações observadas no estudo anterior daquele grupo de autores^(^ 6 ^)^ e por não acreditar que uma equação universal para a função pulmonar seja possível. Os proponentes da equação universal reconhecem que os dados incluídos provenientes da América Latina são escassos, e que aquela equação não deveria ser usada no continente. 

Entretanto, valores para caucasianos foram sugeridos, e testamos a hipótese de que tais valores poderiam se ajustar a nossa população. 

## Métodos

Os valores previstos para caucasianos propostos pela ERS *Global Lung Function Initiative* (GLI) ^(^ 19 ^,^ 21 ^)^ foram calculados para os sexos masculino e feminino, utilizando-se dados de sexo, estatura e idade encontrados em um estudo com valores de referência para a população brasileira.^(^ 16 ^)^ Os pacientes selecionados preencheram um questionário respiratório padronizado,^(^ 22 ^)^ eram não fumantes e assintomáticos respiratórios e não apresentavam doenças cardiopulmonares. Na amostra brasileira foram incluídos 270 indivíduos do sexo masculino (idade entre 25 e 86 anos e estatura entre 152 e 192 cm) e 373 indivíduos do sexo feminino (idade entre 20 e 85 anos e estatura entre 137 e 182 cm).

As equações derivadas para o sexo masculino foram as seguintes^(^ 16 ^)^: 

*CVF* = *E* × 0,0517 −* I *× 0,0207 − 3,18 (limite inferior da normalidade [LIN] = −0,90) 

*VEF*_1 _= *E* × 0,0338 −* I *× 0,0252 − 0,789 (LIN = −0,76) 

*VEF*_1_ */CVF* × 100 = 120,3 − *E* × 0,175 −* I *× 0,197 (LIN = −7,6) 

onde *E* é a estatura em cm e* I *é a idade em anos.

As equações derivadas para o sexo feminino foram as seguintes^(^ 16 ^)^: 

*CVF* = *E* × 0,041 −* I *× 0,0189 − 2,848 (LIN = −0,64) 

*VEF*_1 _= *E* × 0,0314 −* I *× 0,0203 − 1,353 (LIN = −0,61) 

*VEF*_1_ */CVF* × 100 = 111,5 − *E* × 0,140 −* I *× 0,158 (LIN = −8,3) 

A equação a partir do estudo GLI deriva os parâmetros através da seguinte equação: 

log(*Y*) = 5*a* + *b* × log(*H*) + *c* × log(*A*) + *AS* + *d* × grupo 

onde *Y* é a variável dependente, *H* é a estatura em cm, *A* é a idade em anos, e *AS* é *age-spline* (variável interpolante para a idade). 

O grupo assume o valor de 1 para os caucasianos, que foi utilizado no presente estudo. A equação brasileira para CVF e VEF_1_ é linear: 

*Y* = *a* × *E* − *b* ×* I *− constante 

Os valores médios encontrados na amostra brasileira para CVF, VEF_1_, VEF_1_/CVF e seus limites inferiores foram comparados com os valores previstos a partir do GLI, calculados com base na idade e na estatura individual da amostra brasileira. As comparações foram feitas por teste de t pareado. 

A seguir, tomando por base os valores brasileiros, foi feita uma regressão linear entre a idade como variável independente e a estatura como variável dependente. Pelas equações de regressão, sendo os valores de r significantes, foi calculado o valor esperado para a estatura para os valores de idade de 25, 50 e 75 anos em ambos os sexos. Para essas combinações, os valores derivados para as equações GLI e brasileiras foram tabulados e comparados.

Todos os procedimentos estatísticos foram realizados pelo programa IBM SPSS Statistics, versão 20.0 (IBM Corp., Armonk, NY, EUA).

## Resultados

As diferenças das médias dos valores encontrados nas equações brasileiras e dos valores previstos nas equações da GLI, incluindo os limites inferiores, são mostradas na Tabela 1. No sexo masculino, os valores encontrados para todas as variáveis estudadas foram significativamente maiores na amostra brasileira que os previstos pelas equações da GLI. No sexo feminino, tais diferenças foram praticamente nulas, exceto para o limite inferior da relação VEF_1_/CVF, para a qual os valores brasileiros foram significativamente maiores.

**Tabela 1:** Diferenças das médias das variáveis estudadas por sexo, calculadas pela subtração dos valores previstos encontrados na população brasileira(16) daqueles previstos pelas equações da Global Lung Function Initiative.(19,21)a

Variáveis	Sexo					
	Masculino			Feminino		
	Δ	t	p	Δ	t	p
CVF	0,29 ± 0,62	7,81	< 0,001	−0,01 ± 0,38	−0,75	0,46
LI	0,30 ± 0,59	9,41	< 0,001	0,01 ± 0,38	0,65	0,52
VEF_1_	0,28 ± 0,50	9,06	< 0,001	0,00 ± 0,33	0,36	0,72
LI	0,29 ± 0,48	10,12	< 0,001	−0,02 ± 0,33	−0,93	0,36
VEF_1_/CVF	0,93 ± 4,89	3,14	0,002	0,02 ± 5,00	0,06	0,95
LI	3,27 ± 4,71	11,43	< 0,001	3,68 ± 5,23	13,55	0,001

LI : limite inferior

a Valores expressos em média ± dp.

Quando os dados tabulados para as diversas combinações de idade e estatura foram comparados, dados adicionais puderam ser observados (Tabelas 2 e 3). Nos homens, as diferenças se tornaram mais evidentes em indivíduos com menor estatura e maior idade. Para a CVF, em indivíduos com 75 anos, as diferenças para CVF e limite inferior da CVF foram de 0,36 L e 0,38 L, respectivamente. Para esses mesmos indivíduos, a diferença de VEF_1_ e de seu limite inferior foi de 0,29 L para ambos.

**Tabela 2:** Comparação entre valores espirométricos previstos para a população brasileira(16) e aqueles previstos pelas equações da Global Lung Function Initiative (GLI)(19,21) para combinações de idade e estatura em indivíduos do sexo masculino.

Variáveis	Idade, anos	Altura, cm	Pereira et al.^(16)^	GLI^(19,21)^
CVF, L	25	175	5,35	5,18
	50	170	4,58	4,48
	75	165	3,80	3,44
LI	25	175	4,45	4,19
	50	170	3,68	3,50
	75	165	2,90	2,52
VEF_1_, L	25	175	4,50	4,35
	50	170	3,69	3,56
	75	165	2,90	2,61
LI	25	175	3,74	3,51
	50	170	2,93	2,78
	75	165	2,14	1,85
VEF_1_/CVF	25	175	0,85	0,85
	50	170	0,81	0,80
	75	165	0,77	0,76
LI	25	175	0,77	0,73
	50	170	0,73	0,69
	75	165	0,69	0,62

LI : limite inferior.

**Tabela 3:** Comparação entre valores espirométricos previstos para a população brasileira(16) e aqueles previstos pelas equações da Global Lung Function Initiative (GLI)(19,21) para combinações de idade e estatura em indivíduos do sexo feminino.

Variáveis	Idade, anos	Altura, cm	Pereira et al.^(16)^	GLI^(19,21)^
CVF, L	25	162	3,82	3,84
	50	158	3,18	3,24
	75	153	2,47	2,31
LI	25	162	3,18	3,07
	50	158	2,54	2,53
	75	153	1,83	1,63
VEF_1_, L	25	162	3,23	3,30
	50	158	2,60	2,60
	75	153	1,90	1,79
LI	25	162	2,62	2,65
	50	158	1,93	2,03
	75	153	1,32	1,28
VEF_1_/CVF	25	162	0,85	0,87
	50	158	0,81	0,81
	75	153	0,78	0,78
LI	25	162	0,77	0,75
	50	158	0,73	0,70
	75	153	0,68	0,64

LI : limite inferior.

Chama também a atenção o menor valor da relação VEF_1_/CVF prevista por GLI, sendo essa diferença acentuada com a idade. 

## Discussão

No presente estudo, a equação universal proposta para valores espirométricos se mostrou incapaz de predizer com precisão esses valores na população brasileira.

Diversas equações para valores de referência foram publicadas nas últimas décadas. Os valores esperados para indivíduos com uma dada combinação de idade e estatura podem diferir consideravelmente.^(^ 1 ^-^ 3 ^)^

Tais variações podem ser explicadas pelos critérios de seleção das populações 'normais', pelos equipamentos usados, pelas técnicas de medição, pela variabilidade biológica das populações, por fatores socioeconômicos e ambientais, assim como pelos modelos estatísticos utilizados na análise dos dados.

Em 2005, uma diretriz sobre função pulmonar foi publicada conjuntamente pela ATS e ERS.^(^ 1 ^)^ Valores de referência a serem adotados foram sugeridos para crianças e adultos norte-americanos; porém, valores para outros locais foram deixados em aberto. Essa falta de recomendação levou um grupo de autores, liderados por Quanjer, a fundar a GLI em Berlim em 2008. Em abril de 2010, o grupo recebeu, à semelhança do anteriormente ocorrido,^(^ 5 ^,^ 6 ^)^ a chancela da ERS como uma força-tarefa.^(^ 19 ^)^ Em 2012, valores derivados de dados enviados de diversos locais foram, à semelhança do ocorrido em 1993 com dados europeus,^(^ 6 ^)^ agrupados, e valores de referência para indivíduos de 3 a 95 anos foram sugeridos. No total, 74.187 indivíduos não fumantes de 26 países de cinco continentes foram incluídos em equações derivadas pela combinação de diversos estudos. Os dados relativos à América do Sul, derivados de um estudo latino-americano^(^ 20 ^)^ e de uma amostra de crianças no México,^(^ 23 ^)^ foram desconsiderados por diferenças na estatura e nos valores previstos, além da falta de dados para indivíduos entre 25 e 40 anos. Entretanto, pelo suplemento publicado, 178 casos de brasileiros caucasianos foram incluídos.^(^ 19 ^)^

Os valores para caucasianos foram derivados especialmente de cinco grandes estudos: dois norte-americanos^(^ 24 ^,^ 25 ^)^ e três europeus.^(^ 7 ^,^ 10 ^,^ 13 ^)^ É interessante notar que os valores derivados por aqueles estudos diferem, o que foi atribuído aos diferentes equipamentos utilizados. Entretanto, diversos fatores, tais como seleção da amostra, técnicas de medição e controle de qualidade também influenciam os resultados obtidos, o que complica a agregação de diferentes estudos.

Comparando a equação GLI com os dados derivados de uma amostra brasileira utilizada para derivação de valores de referência,^(^ 16 ^)^ nós observamos que tal equação resulta em valores menores no sexo masculino, tanto para os valores previstos quanto para seus limites inferiores. No sexo feminino, os valores são bastante semelhantes, exceto quanto à relação VEF_1_/CVF e seu limite inferior, maiores na amostra brasileira. Esses achados indicam que a utilização da equação GLI irá deixar de diagnosticar reduções da CVF e, portanto, terá menor sensibilidade na detecção de distúrbios restritivos no sexo masculino. Em ambos os sexos, a sensibilidade para o diagnóstico de distúrbio obstrutivo será menor com o uso das equações da GLI, visto que o limite inferior para a relação VEF_1_/CVF é significativamente menor, especialmente em indivíduos com idade mais avançada.

As diferenças entre os valores previstos para a relação VEF_1_/CVF e os limites inferiores calculados pela equação da GLI são variáveis devido ao modelo de regressão aplicado; porém, são, em média, de 0,11 para o sexo masculino e de 0,12 para o sexo feminino,^(^ 21 ^)^ o que excede os valores derivados no Brasil (0,08 no sexo masculino e 0,09 no sexo feminino).^(^ 16 ^)^

Estudos recentes compararam o diagnóstico espirométrico pela equação sugerida pelo GLI e outras. Um estudo comparou os diagnósticos espirométricos por três equações em 17.572 testes de indivíduos de 18-85 anos de idade provenientes de laboratórios na Austrália e na Polônia.^(^ 26 ^)^ As equações derivadas do GLI mostraram valores superiores àquelas derivadas por Quanjer et al.,^(^ 6 ^)^ como já esperado. Diferenças nos limites inferiores resultaram em uma redução significativa no diagnóstico de distúrbio restritivo quando a equação GLI foi comparada à equação NHANES III, embora essa tenha sido incorporada à equação GLI (mas constituindo menos de 4% da amostra). O distúrbio restritivo foi diagnosticado em 22,6% pela equação NHANES III em comparação a 17,1% pela equação GLI, no sexo masculino. No sexo feminino, as proporções foram 22,8% e 8,1%, respectivamente.

Em outro estudo realizado na Tunísia, valores previstos localmente e aqueles sugeridos pelo GLI foram aplicados em 1.192 espirometrias consecutivas em adultos de 18-60 anos.^(^ 27 ^)^ Novamente, o diagnóstico de distúrbio restritivo foi realizado em maior proporção pelo uso da equação local (19,0%) em comparação à derivada pelo GLI (8,4%).

Os achados dos estudos acima não são surpreendentes, dado o largo intervalo para a determinação dos limites inferiores pela equação GLI, resultado da junção de diversas equações com controle de qualidade e resultados diferentes.

Em conclusão, os valores sugeridos pela equação multiétnica sugerida pela GLI, derivada para caucasianos, diferem significativamente dos valores derivados para indivíduos caucasianos do sexo masculino no Brasil. No sexo feminino, os valores derivados são semelhantes para CVF, VEF_1_ e seus limites inferiores. Para ambos os sexos, o limite inferior da relação VEF_1_/CVF é significativamente menor pela equação GLI.
